# Autoantibody profiling to predict response to the anti-PD-1 therapy, pembrolizumab, in rare tumors

**DOI:** 10.1016/j.esmoop.2025.105518

**Published:** 2025-08-05

**Authors:** M.H. Derbala, J. Hajjar, B. Stephen, S.A. Gurses, E. Kwiatkowski, P. Budde, H.-D. Zucht, M. Bräutigam, A.-S. Lindemann, B.A. Abhari, M.A. Gouda, L. Castillo, A. Zarifa, J.A. How, E. Rodriguez, J.T. Moyers, D.S. Hong, S. Fu, F. Meric-Bernstam, A. Naing

**Affiliations:** 1 Department of Investigational Cancer Therapeutics, The University of Texas MD Anderson Cancer Center, Houston, USA; 2 Division of Immunology, Allergy & Retrovirology, William T. Shearer Center for Human Immunobiology, Baylor College of Medicine, Texas Children’s Hospital, Houston, USA; 3 Department of Biostatistics and Data Science, UT Health Houston, Houston, USA; 4 Oncimmune Germany GmbH, Dortmund, Germany; 5 Endeavor Health, Chicago, USA; 6 Department of Gynecologic Oncology & Reproductive Medicine, The University of Texas MD Anderson Cancer Center, Houston, USA; 7 Angeles Clinic and Research Institute, Cedars-Sinai Affiliate, Los Angeles, USA

**Keywords:** autoantibodies, predictive biomarkers, immune checkpoint inhibitors, cancer immunotherapy, rare tumors

## Abstract

**Background:**

Immune checkpoint inhibitors (ICIs) have limited response rates in selected patients and can cause potentially life-threatening immune-related adverse events (irAEs). This underscores the urgent need for the development of biomarkers predictive of ICI response. Pre-existing autoantibodies (AAbs) have been linked with responses to ICIs and the development of irAEs. We conducted AAb profiling to assess associations between baseline AAbs and clinical benefit (CB) in patients with rare tumors treated with anti-programmed cell death protein 1-based therapy.

**Patients and methods:**

We conducted AAb profiling using Oncimmune’s SeroTag multiplex antibody discovery technology and immune oncology array containing 1168 antigens. The study included 41 patients with rare tumors who received pembrolizumab compared with healthy controls. We carried out a significance analysis of microarrays to identify baseline AAbs present in these cancer patients and associations between these AAbs and CB (defined as complete response, partial response, or stable disease for ≥6 months). We also investigated associations between baseline AAbs and time to progression (TTP) and overall survival (OS) using Cox proportional hazards and Kaplan–Meier models.

**Results:**

Compared with healthy controls, patients with rare tumors had significantly higher levels of several AAbs at baseline, including those against interferon alpha antigens. Ten patients (24%) had CB. Several AAbs, including those targeting DNA ligase III (LIG3), were associated with CB, TTP, and OS.

**Conclusion:**

The predictive potential of AAbs as biomarkers of ICI therapy is promising. Further evaluation in larger cohorts is needed to validate our findings and elucidate the underlying mechanism.

## Highlights

• Patients with rare tumors have higher pre-treatment autoantibodies against crucial antigens, such as interferon alpha.
• Autoantibodies to DNA ligase III and BIRC6 are associated with favorable treatment outcomes.
• Autoantibody profiling might help personalize cancer therapy by stratifying patients likely to respond to ICIs.

## Introduction

Immune checkpoint inhibitors (ICIs) elicit responses in only a subset of patients,^1^ and their expanded use is increasing the frequency of immune-related adverse events (irAEs).^2^ Thus, there is an urgent need to identify biomarkers that are predictive of ICI response.

Autoantibodies (AAbs) are traditionally associated with autoimmune conditions and play a pivotal role in their prognosis and diagnosis.^3^ Although little is known about the role of the humoral immune system in cancer, a growing body of evidence has linked the humoral immune system with cancer pathogenesis, progression, and treatment response.^4^^,^^5^ AAb signatures have been detected in lung,^6^ ovarian,^7^ gastrointestinal,^8^ prostate,^9^ breast,^10^ liver,^11^ and urinary bladder cancers.^12^^,^^13^ Recent studies have also shown that pre-existing AAbs are linked with ICI therapy response in patients with urothelial cancers^13^ and with the development of irAEs in patients with solid tumors receiving ICIs.^14^ Other studies have shown that AAbs can serve as early indicators of the immune system’s response to a developing tumor, as they are often detected long before any clinical signs of the primary tumor emerge.^4^ Therefore, AAb profiling, which can consolidate immune recognition of diverse cancer-related proteins into a single tool, presents a promising strategy for cancer diagnosis and prognosis.

In this study, we aimed to characterize the AAb profiles of patients with rare malignancies and identify associations between these AAbs and clinical benefit (CB), time to progression (TTP), and overall survival (OS) in the context of treatment with ICIs.

## Patients and methods

### Sex as a biological variable

Our study included both men and women.

### Study design

The study included 41 patients who were enrolled in a phase II study of pembrolizumab in rare cancers conducted at The University of Texas MD Anderson Cancer Center (NCT02721732). The protocol was approved by the Food and Drug Administration (FDA) and the Institutional Review Board (IRB) at The University of Texas MD Anderson Cancer Center. Plasma samples collected before the administration of the anti-programmed cell death protein 1 (anti-PD-1) therapy, pembrolizumab, were retrieved from the MD Anderson biobank. Plasma and serum samples from 53 age- and sex-matched healthy controls (HCs) were provided by the Bavarian Red Cross and BioIVT.

All patients received pembrolizumab at a dose of 200 mg intravenously on day 1 of each 21-day dosing cycle until disease progression or unacceptable toxicity, death, withdrawal of consent, discontinuation from the study treatment for any other reason, or completion of 24 months of treatment with pembrolizumab. Tumor imaging was performed at baseline and after three cycles (9 weeks). Response to treatment was assessed using immune-related Response Evaluation Criteria in Solid Tumors (irRECIST).^15^

We conducted AAb profiling to explore the relationship between baseline AAb levels and treatment outcomes, namely CB, TTP, and OS. CB was defined as complete response (CR), partial response (PR), or stable disease (SD) for ≥6 months as assessed by irRECIST. Given the rarity of these tumors and the significance of SD as a potential indicator of antitumor activity and clinically meaningful treatment effect in patients treated with immunotherapy,^16^ we defined CB as patients who had CR, PR, or SD lasting ≥6 months, as assessed by irRECIST, consistent with the criteria used in previous studies.^17^^,^^18^ TTP was defined as the period from the initiation of the anti-PD-1, pembrolizumab, until disease progression as determined by radiographic imaging or clinical examination. OS was defined as the interval from the initiation of the anti-PD-1, pembrolizumab, until death from any cause. Positive associations were defined as associations between baseline AAbs and favorable treatment outcomes, whereas negative associations were defined as those between baseline AAbs and poor treatment outcomes.

### Statistical analysis

AAb profiling was conducted in accordance with an established protocol.^19^ Utilizing SeroTag multiplex-based technology, we detected immunoglobulin G reactivity against 1168 antigens, with values expressed as median fluorescence intensity (MFI). Low-reactivity antigens (max log2 MFI < 10.5) were filtered out, resulting in 879 antigens. We conducted a significance analysis of microarrays (SAM) to analyze the association of baseline AAbs with CB. AAb biomarker candidates were discovered based on filtering for SAM hits with *P* < 0.05, d-score (D) > 2. The d-score reflects the strength of the association between AAbs and the outcome, adjusted for variance. In the heatmap, samples appear in columns and antigens, which are sorted according to unsupervised hierarchical clustering using the Manhattan distance formula, appear in rows. A Cox proportional hazards model and Kaplan–Meier (KM) estimator were applied to discover associations between baseline AAbs and TTP or OS [Cox regression: unadjusted *P* < 0.05, log2 hazard ratio (HR) ≥ 0.58; KM log-rank test: unadjusted *P* < 0.05]. In the KM analyses of patients with rare tumors, the 25% of patients with the highest MFI for each AAb were compared with the 75% of patients with the lowest MFI for each AAb.

### Study approval

The study obtained ethics approval from the IRB of the University of Texas MD Anderson Cancer Center [Federalwide Assurance Number (FWA): 00000363 and Office of Human Research Protection (OHRP) IRB Registration Number: IRB00000121]. The study was conducted in accordance with the Declaration of Helsinki and the International Conference on Harmonization Good Clinical Practice guidelines. All the study participants provided written informed consent before enrollment and for the use of their biospecimens and clinical data for research purposes. Samples from HCs were provided by the Bavarian Red Cross (Munich, Germany) and BioIVT (Burgess Hill, UK).

## Results

### Patient population

The 41 patients’ demographic characteristics are provided in Table 1. Most patients (54%) were women. The median age was 66 years. The most common histological tumor type was carcinoma of unknown primary (32%), followed by squamous-cell carcinoma of the skin (22%). Of the 41 patients, 10 (24%) had CB, whereas 27 (66%) had SD for <6 months or progressive disease and 4 (10%) were non-assessable for response. All patients in our study were immunotherapy-naive before receiving an anti-PD1-based treatment regimen at our institution. For all 41 patients, the median TTP and OS duration were 2.3 months and 8.6 months, respectively.

**Table 1:** Patients’ characteristics

Characteristic	No. of patients (%)^a^ (*n* = 41)
Age, median (range), years	66 (35-84)
Sex	
Male	19 (46)
Female	22 (54)
Tumor type	
Carcinoma of unknown primary	13 (32)
Squamous-cell carcinoma of the skin	9 (22)
Small-cell malignancy of non-pulmonary origin	7 (17)
Adrenocortical carcinoma	6 (15)
Vascular sarcoma	5 (12)
Other rare histology	1 (2)
Treatment response	
CR, PR, or SD ≥6 months^b^	10 (24)
SD <6 months or PD	27 (66)
Not assessable	4 (10)

CR, complete response; PD, progressive disease; PR, partial response; SD, stable disease.

a Unless otherwise indicated.

b Patients with these responses were considered to have clinical benefit.

### Autoantibodies in cancer patients versus healthy controls

**Figure 1:**
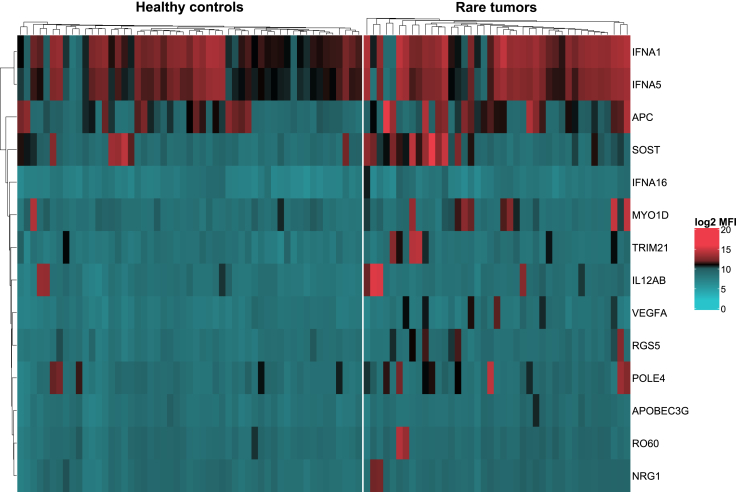
**Heatmap of positive autoantibodies in patients with rare tumors versus healthy controls.** The heatmap depicts candidate antigens that elicited high levels of autoantibody responses in patients with rare tumors versus healthy controls. Samples appear in columns and antigens, which are sorted according to unsupervised hierarchical clustering using the Manhattan distance formula, appear in rows.

SAM identified 14 baseline AAbs that were significantly elevated in patients with rare tumors compared with HCs. These AAbs included AAbs against interferon-α (IFNA) antigens, including IFNA1 [*P* = 0.002, D = 2.49, fold-change (FC) = 1.8], IFNA5 (*P* = 0.004, D = 2.29, FC = 1.6), IFNA16 (*P* = 0.009, D = 2.06, FC = 1.4); neuregulin 1 (NRG1; *P* = 0.001, D = 2.84, FC = 1.48); tripartite motif-containing 21 [TRIM21 (also known as Ro52); *P* = 0.001, D = 2.75, FC = 1.7]; adenomatous polyposis coli (APC; *P* = 0.002, D = 2.52, FC = 1.9); and vascular endothelial growth factor A (VEGFA; *P* = 0.004, D = 2.28, FC = 1.5) (Figure 1 and Supplementary Table S1, available at https://doi.org/10.1016/j.esmoop.2025.105518).

### Association between autoantibodies and treatment outcomes

**Figure 2:**
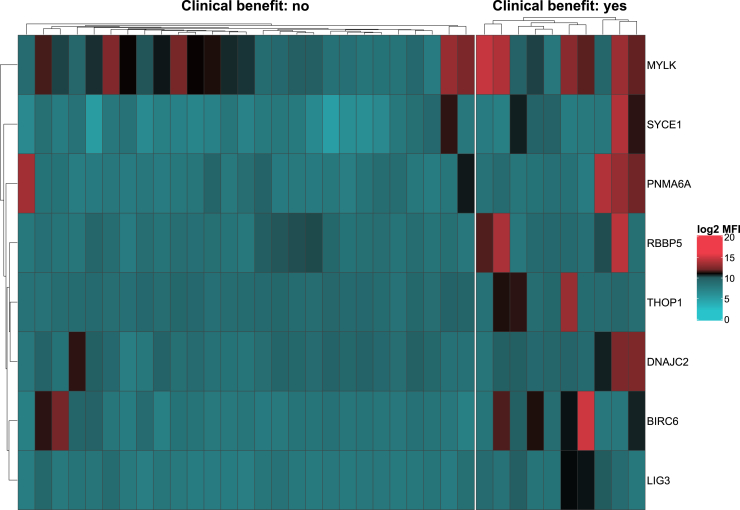
**Heatmap of autoantibodies associated with clinical benefit.** The heatmap depicts antigens that were associated with clinical benefit in patients with rare tumors. Samples appear in columns and antigens appear in rows.

Eight baseline AAbs had significant positive associations with CB; these included AAbs against DNA ligase 3 (LIG3; *P* = 0.001, D = 4.07, FC = 2.5), baculoviral inhibitor of apoptosis repeat-containing protein 6 (BIRC6; *P* = 0.002, D = 2.97, FC = 3.5), and synaptonemal complex central element protein 1 [SYCE1 (also known as cancer/testis antigen 76); *P* = 0.002, D = 2.96, FC = 3.9] (Figure 2 and Supplementary Table S2, available at https://doi.org/10.1016/j.esmoop.2025.105518). BIRC6 and LIG3 were both associated with improved TTP, and LIG3 was additionally linked to better OS, highlighting these markers as consistent indicators of favorable outcomes.

**Figure 3:**
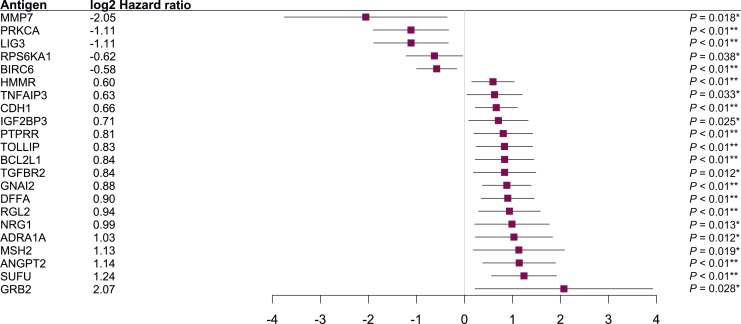
**Autoantibodies positively and negatively associated with time to progression.** Log2 hazard ratios (indicated by squares), 95% confidence intervals (indicated by horizontal lines), and *P* values were from a Cox proportional hazards model. ∗ *P* value <0.05; ∗∗*P* value <0.01.

Twenty-two baseline AAbs had significant associations with TTP (Figure 3 and Supplementary Table S3, available at https://doi.org/10.1016/j.esmoop.2025.105518). Among these, 5 AAbs, including those against matrix metalloproteinase 7 (MMP7; HR = 0.24, *P* = 0.018), protein kinase C alpha (PRKCA; HR = 0.46, *P* = 0.006), LIG3 (HR = 0.46, *P* = 0.005), and BIRC6 (HR = 0.67, *P* = 0.007) were significantly associated with increased TTP, whereas 17 AAbs, including those against growth factor receptor bound protein 2 (GRB2; HR = 4.2, *P* = 0.028) and suppressor of fused homolog (SUFU; HR = 2.36, *P* < 0.0005), were associated with significantly shorter TTP (Figure 3 and Supplementary Figure S1 and Table S3, available at https://doi.org/10.1016/j.esmoop.2025.105518).

**Figure 4:**
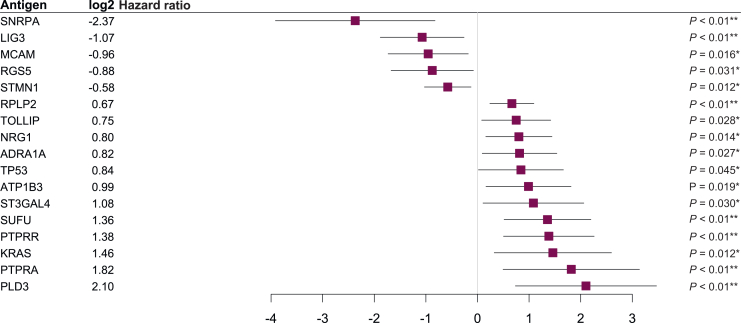
**Autoantibodies positively and negatively associated with overall survival.** Log2 hazard ratios (indicated by squares), 95% confidence intervals (indicated by horizontal lines), and *P* values were from a Cox proportional hazards model. ∗*P* value <0.05; ∗∗*P* value <0.01.

Additionally, 17 baseline AAbs were significantly associated with OS (Figure 4 and Supplementary Table S4, available at https://doi.org/10.1016/j.esmoop.2025.105518). Of these, 5 AAbs, including those against small nuclear ribonucleoprotein polypeptide A (SNRPA; HR = -0.19, *P* = 0.003), LIG3 (HR = 0.48, *P* = 0.01), and melanoma cell adhesion molecule (MCAM; HR = 0.52, *P* = 0.016), were significantly associated with longer OS, whereas 12 AAbs, including those against phospholipase D family member 3 (PLD3; HR = 4.29, *P* = 0.003), SUFU (HR = 2.56, *P* = 0.002), proto-oncogene KRAS antigen (aa 1-164, G12D and Q61H mutations; HR = 2.75, *P* = 0.012), and tumor suppressor TP53 antigen (HR = 1.79, *P* = 0.045) were significantly associated with shorter OS (Figure 4 and Supplementary Figure S2 and Table S4, available at https://doi.org/10.1016/j.esmoop.2025.105518).

Of note, AAbs against SUFU, NRG1, protein tyrosine phosphatase receptor type R (PTPRR), adrenoceptor alpha 1A (ADRA1A), and Toll-interacting protein (TOLLIP) were significantly associated with both shorter TTP and shorter OS (Figures 3 and 4; Supplementary Tables S3 and S4, available at https://doi.org/10.1016/j.esmoop.2025.105518). Conversely, only anti-LIG3 AAbs were significantly associated with both longer TTP and longer OS (Figures 3 and 4; Supplementary Tables S3 and S4, available at https://doi.org/10.1016/j.esmoop.2025.105518) suggesting that these autoantibodies may serve as markers of poorer prognosis.

## Discussion

To our knowledge, this is the first study to explore the AAb profiles in patients with rare tumors, a group who are largely underexplored, particularly in the context of ICI therapy. Using an advanced multiplex-based immunoassay with a wide repertoire of antigens, we investigated the intricate landscape of AAbs in patients with rare tumors and identified associations between AAbs and treatment outcomes such CB, TTP, and OS. While exploratory, our findings provide insight into the role of autoantibodies in cancer biology and treatment outcomes.

Our study revealed that, compared with age- and sex-matched HCs, patients with rare tumors had significantly higher levels of AAbs against IFNA antigens. Anti-interferon AAbs have been described in the context of infections, autoimmune diseases, thymoma, and even in some healthy individuals, where they can neutralize type-I interferon activity.^20^ The IFNA family is integral to type-I interferon signaling, which has a multifaceted role in cancer biology.^21^^,^^22^ While type-I interferon serves as a key mediator of inflammatory signals, its chronic activation, particularly in the cancer escape phase, may induce feedback suppression, presenting a dichotomous influence on immune response and cancer progression.^21^^,^^22^ Similarly, patients with rare tumors had higher levels of anti-TRIM21 AAbs than HCs did. TRIM21, an antibody-binding protein involved in regulating type-I interferon responses, plays complex roles in cancer.^23^ Depending on the type of cancer, TRIM21 can act as either an oncogene or a tumor suppressor. In patients with pancreatic and thyroid cancers, TRIM21 overexpression has been associated with poor prognosis, whereas in patients with diffuse large B-cell lymphoma and ovarian, gastric, and breast cancers, low TRIM21 levels have been linked to negative clinical outcomes.^24^ However, unlike their established role in autoimmune diseases, the role of anti-TRIM21 Abs in various cancers remains unclear.^25^ In patients with esophageal squamous-cell carcinoma, anti-TRIM21 Abs have been linked to poor survival, whereas in those with ovarian cancer, a high prevalence of these Abs has been associated with improved OS.^24^ These seemingly conflicting data raise questions about whether anti-TRIM21 Abs interfere with TRIM21’s function or merely indicate its hyper-expression, which has to be further investigated.^24^ Additionally, compared with HCs, patients with rare tumors also had higher levels of AAbs against NRG1, APC, and VEGFA. Although the proteins they target are known to be expressed in different cancers,^26^, ^27^, ^28^, ^29^ these AAbs’ translational and clinical potential should be investigated further to determine whether they develop to counteract oncogenes and mutated suppressor genes or contribute to the development of therapeutic antibodies.

Our findings also show that high levels of anti-LIG3 AAbs were significantly associated with CB, TTP, and OS (Figure 2; Supplementary Tables S2-S4, available at https://doi.org/10.1016/j.esmoop.2025.105518). LIG3 is a DNA repair protein, and its expression has been linked with longer relapse-free survival in patients with breast cancer^30^; however, the prognostic utility of anti-LIG3 AAbs remains unexplored and should be validated in future studies. Notably, one recent preclinical study in mice showed that depleting LIG3 renders BRCA1-deficient mammary tumors sensitive to poly (ADP-ribose) polymerase (PARP) inhibitors.^31^ Whether a similar effect occurs in patients with rare tumors who receive anti-PD-1 therapy and have CB remains uncertain and warrants further investigation. In addition, some recent studies demonstrated that mutations in DNA repair genes are associated with improved OS in ICI-treated patients, regardless of the tumor mutation burden.^32^, ^33^, ^34^ As it is widely believed that genetic mutations might trigger AAb production,^35^ this could be one possible explanation for the presence of anti-LIG3 AAbs in our patients and their association with favorable clinical outcomes. Similar to anti-LIG3 AAbs, high levels of anti-BIRC6 AAbs were significantly associated with CB and longer TTP (Figure 3; Supplementary Tables S2 and S3, available at https://doi.org/10.1016/j.esmoop.2025.105518). Prior studies in patients with non-small-cell lung cancer (NSCLC) revealed that the increased expression of BIRC6, an inhibitor of apoptosis family members, was correlated with poor 3-year relapse-free survival and with metastasis and predicted resistance to chemotherapy.^36^ Moreover, upon the silencing of the *BIRC6* gene, NSCLC cells became sensitive to cisplatin.^36^ Another study in a patient-derived xenograft model of castration-resistant prostate cancer also demonstrated that high BIRC6 protein levels were associated with resistance to the androgen receptor inhibitor enzalutamide and that targeting BIRC6 inhibited cancer growth both *in vitro* and *in vivo*. Yet, much remains to be discovered regarding the mechanism behind the relationship between the increased anti-BIRC6 AAb levels and CB we observed in patients with rare tumors in the present study. We hypothesize that the elevated AAb level is an adaptive response elicited to neutralize the BIRC6 protein, given the latter’s anti-apoptotic effects.

We also found that anti-MMP7 AAbs were associated with improved TTP (Supplementary Table S3 and Figure S1, available at https://doi.org/10.1016/j.esmoop.2025.105518). MMP7 has been recognized as an oncogenic protein that contributes to the tumorigenesis, infiltration, and metastasis of different cancers.^37^ Previous studies showed that patients diagnosed with gastric,^37^ esophageal,^37^ and non-melanoma skin cancers^38^ had elevated concentrations of anti-MMP7 AAbs, highlighting the prospect of using these AAbs as a screening biomarker.^37^ However, there is a paucity of data regarding the value of anti-MMP7 Abs as prognostic and/or predictive biomarkers in cancer patients; only one study in patients with oral squamous-cell carcinoma identified anti-MMP7 Abs to be an independent predictor of poor OS.^39^ On the contrary, anti-GRB2 AAbs were associated with poor TTP in our cohort of patients with rare tumors (Supplementary Table S3 and Figure S1, available at https://doi.org/10.1016/j.esmoop.2025.105518). GRB2 is an adaptor protein that plays a critical intermediary role in tumorigenic signaling pathways.^40^ Prior studies revealed that increased GRB2 expression is correlated with poor prognosis in patients with esophageal^40^ and gastric cancers.^41^ Furthermore, in patients with prostate cancer, GRB2 overexpression was linked to shorter recurrence-free survival.^42^ As such, it is unclear why anti-GRB2 AAbs were associated with a higher risk of progression in the present study. A single AAb might have inadequate prognostic or predictive capacity, but leveraging a panel of AAbs could mitigate this problem. We also noted that AAbs against the G protein subunit alpha I2 (GNAI2) were associated with shorter TTP, a finding that aligns with a recent study in urothelial cancer,^13^ where anti-GNAI2 was correlated with poor progression-free survival. No other AAbs were common between our study and that of urothelial cancer, which may be due to differences in the scope of tumor types studied.

We also observed that anti-SNRPA AAbs were associated with longer OS (Supplementary Table S4, available at https://doi.org/10.1016/j.esmoop.2025.105518). SNRPA is a component of the U1 small nuclear ribonucleoprotein complex, and its expression is correlated with poor outcomes in patients with gastric cancer.^43^ In addition, higher levels of SNRPA have been associated with both shorter OS and shorter recurrence-free survival in patients with hepatocellular carcinoma.^44^ Contrarily, anti-PLD3 AAbs were significantly associated with poor OS (Supplementary Table S4 and Figure S2, available at https://doi.org/10.1016/j.esmoop.2025.105518). PLD3, a member of the phosphodiesterase family, is believed to play a role in cancer development and progression.^45^^,^^46^ Studies of the utilization of PLD3 as a prognostic biomarker are sparse. One study developed a risk prediction model utilizing a panel of five AAb biomarkers, including anti-PLD3 AAbs, for the early diagnosis of small-cell lung cancer.^47^ Further research is warranted to fully unravel the clinical significance of both anti-SNRPA and anti-PLD3 AAbs and their potential implications in different cancer patient populations.

Further, we noted that five AAbs (SUFU, ADRA1A, PTPRR, NRG1, and TOLLIP) were associated with both shorter TTP and OS (Supplementary Tables S3 and S4, available at https://doi.org/10.1016/j.esmoop.2025.105518). SUFU negatively regulates the hedgehog signaling pathway, which plays a role in modulating PD-L1 expression and T-cell activation.^48^ SUFU expression has been associated with poor OS and disease-free survival rates in patients with colorectal cancer^49^ and aggressive glioma.^50^ However, additional research is needed to further elucidate the potential biomarker role of anti-SUFU AAbs and the other AAbs exhibiting varying associations with the clinical outcomes investigated in our study.

In this study we identified autoantibodies associated with outcome which bind to intracellular proteins (e.g. TP53, LIG3, BIRC6) as well as to membrane-associated proteins (ADRA1A, NRG1, MCAM, TGFBR2, and HMMR) and extracellular proteins (MMP7 and ANGPT2). Although intracellular proteins are not typically accessible to autoantibodies under normal physiological conditions, tumor cells undergoing stress or cell death can release intracellular contents, allowing these antigens to be recognized by the immune system.^51^, ^52^, ^53^ By targeting tumor-associated antigens, AAbs can enhance via their constant region (Fc) Fcg receptor (FcgR)-mediated antigen uptake and presentation, thereby promoting additional T-cell priming and antitumor immunity via checkpoint inhibitors.^54^, ^55^, ^56^, ^57^ Furthermore, AAbs binding to membrane-associated antigens may mediate complement or antibody-dependent cellular cytotoxicity (ADCC) via engagement of FcgR expressed on NK cells.^58^, ^59^, ^60^ Depending on the context, AAbs forming immune complexes may contribute to chronic inflammation or immunosuppression leading to lack of response to cancer immunotherapy.^61^^,^^62^

Another group of AAbs may influence cancer progression or metastasis through mechanisms including receptor activation, blockade, or modulating the activity of soluble factors. For example, ADRA1A is a G protein-coupled receptor, which is involved in vasoconstriction and has been implicated in tumor proliferation and angiogenesis.^63^^,^^64^ Anti-ADRA1A autoantibodies have been described in prostate cancer patients and exert functional activity in *in vitro* models supporting carcinogenesis by excessive receptor stimulation.^64^ Another example is the cell adhesion molecule MCAM also known as MUC18 or CD146, which is linked to metastasis and poor prognosis in melanoma and other malignancies. MUC18 has been investigated as a potential therapeutic target.^65^ Monoclonal antibodies targeting MUC18 have shown efficacy in preclinical models of melanoma, where they inhibited tumor growth, reduced metastasis, and suppressed MMP activity.^66^ While we do not yet have functional data for anti-MUC18 AAbs, their detection is promising and suggests potential biological relevance that warrants further investigation.

This study has several limitations, but its findings offer significant insights and pave the way for future exploration. While AAbs are traditionally associated with autoimmune diseases and play a central role in their diagnostic and prognostic evaluation, their involvement in cancer remains an underexplored area. Our research, primarily exploratory in nature, identifies associations between AAbs and treatment outcomes, though it does not delve into the mechanistic underpinnings. The study’s small cohort size reduces statistical power and precludes the inclusion of a training or an independent validation cohort, thereby limiting the generalizability of the findings. However, the inclusion of matched HCs strengthens the validity of our results by providing a reliable comparative baseline. Overall, this study offers a foundation for refining specific AAb targets in future studies. Moreover, our rigorous methodology ensures the reproducibility of our findings, further reinforcing their credibility. Additionally, while the heterogeneous nature of the rare tumors in our cohort poses challenges in drawing definitive conclusions, it also enhances the value of this study. This diversity contributes to broader, pan-cancer analyses and provides important insights into cancer biology. As part of our future research, we plan to evaluate the association between AAb profiles and irAEs, which could enhance our understanding of their role in predicting irAEs.

In conclusion, our study highlights elevated levels of AAbs against IFNA and other antigens implicated in various cancers, as well as associations between AAbs targeting LIG3 and BIRC6 with more favorable treatment outcomes. These findings lay the groundwork for further investigations into the role of AAbs in cancer immunotherapy. Larger cohort studies are needed to validate these results and refine their clinical applications.

## References

[1] Bai R., Lv Z., Xu D., Cui J. (2020). Predictive biomarkers for cancer immunotherapy with immune checkpoint inhibitors. Biomark Res.

[2] Yin Q., Wu L., Han L. (2023). Immune-related adverse events of immune checkpoint inhibitors: a review. Front Immunol.

[3] Dudas S.P., Chatterjee M., Tainsky M.A. (2010). Usage of cancer associated autoantibodies in the detection of disease. Cancer Biomark.

[4] Zaenker P., Gray E.S., Ziman M.R. (2016). Autoantibody production in cancer–the humoral immune response toward autologous antigens in cancer patients. Autoimmun Rev.

[5] Macdonald I.K., Parsy-Kowalska C.B., Chapman C.J. (2017). Autoantibodies: opportunities for early cancer detection. Trends Cancer.

[6] Patel A.J., Tan T.M., Richter A.G., Naidu B., Blackburn J.M., Middleton G.W. (2022). A highly predictive autoantibody-based biomarker panel for prognosis in early-stage NSCLC with potential therapeutic implications. Br J Cancer.

[7] Anderson K.S., Cramer D.W., Sibani S. (2015). Autoantibody signature for the serologic detection of ovarian cancer. J Proteome Res.

[8] Zayakin P., Ancans G., Silina K. (2013). Tumor-associated autoantibody signature for the early detection of gastric cancer. Int J Cancer.

[9] Wang X., Yu J., Sreekumar A. (2005). Autoantibody signatures in prostate cancer. N Engl J Med.

[10] Fernandez-Madrid F., Tang N., Alansari H. (2004). Autoantibodies to annexin XI-A and other autoantigens in the diagnosis of breast cancer. Cancer Res.

[11] Zhang J.Y., Tan E.M. (2010). Autoantibodies to tumor-associated antigens as diagnostic biomarkers in hepatocellular carcinoma and other solid tumors. Expert Rev Mol Diagn.

[12] Castel-Ajgal Z., Goulvestre C., Zaibet S. (2022). Preexisting autoantibodies and immune related adverse events in metastatic urothelial carcinoma patients treated by pembrolizumab. Clin Genitourin Cancer.

[13] Ravi P., Freeman D., Thomas J. (2024). Comprehensive multiplexed autoantibody profiling of patients with advanced urothelial cancer. J Immunother Cancer.

[14] Daban A., Gonnin C., Phan L. (2023). Preexisting autoantibodies as predictor of immune related adverse events (irAEs) for advanced solid tumors treated with immune checkpoint inhibitors (ICIs). Oncoimmunology.

[15] Bohnsack O., Hoos A., Ludajic K. (2014). Adaptation of the immune related response criteria: irRECIST. Ann Oncol.

[16] Wolchok J.D., Hoos A., O’Day S. (2009). Guidelines for the evaluation of immune therapy activity in solid tumors: immune-related response criteria. Clin Cancer Res.

[17] Snyder A., Makarov V., Merghoub T. (2014). Genetic basis for clinical response to CTLA-4 blockade in melanoma. N Engl J Med.

[18] Chen P.L., Roh W., Reuben A. (2016). Analysis of immune signatures in longitudinal tumor samples yields insight into biomarkers of response and mechanisms of resistance to immune checkpoint blockade. Cancer Discov.

[19] Budde P., Zucht H.D., Vordenbaumen S. (2016). Multiparametric detection of autoantibodies in systemic lupus erythematosus. Lupus.

[20] Bastard P., Gervais A., Le Voyer T. (2024). Human autoantibodies neutralizing type I IFNs: from 1981 to 2023. Immunol Rev.

[21] Snell L.M., McGaha T.L., Brooks D.G. (2017). Type I interferon in chronic virus infection and cancer. Trends Immunol.

[22] Cheon H., Wang Y., Wightman S.M., Jackson M.W., Stark G.R. (2023). How cancer cells make and respond to interferon-I. Trends Cancer.

[23] Alomari M. (2021). TRIM21 - a potential novel therapeutic target in cancer. Pharmacol Res.

[24] Hsu C.H., Yu Y.L. (2023). The interconnected roles of TRIM21/Ro52 in systemic lupus erythematosus, primary Sjogren’s syndrome, cancers, and cancer metabolism. Cancer Cell Int.

[25] Jones E.L., Laidlaw S.M., Dustin L.B. (2021). TRIM21/Ro52 - roles in innate immunity and autoimmune disease. Front Immunol.

[26] Nagasaka M., Ou S.I. (2022). NRG1 and NRG2 fusion positive solid tumor malignancies: a paradigm of ligand-fusion oncogenesis. Trends Cancer.

[27] Zhang Y., Liu X., Li A., Tang X. (2022). A pan-cancer analysis on the carcinogenic effect of human adenomatous polyposis coli. PLoS One.

[28] Sopo M., Anttila M., Hamalainen K. (2019). Expression profiles of VEGF-A, VEGF-D and VEGFR1 are higher in distant metastases than in matched primary high grade epithelial ovarian cancer. BMC Cancer.

[29] Yang Y., Cao Y. (2022). The impact of VEGF on cancer metastasis and systemic disease. Semin Cancer Biol.

[30] Sun L., Liu X., Song S. (2021). Identification of LIG1 and LIG3 as prognostic biomarkers in breast cancer. Open Med (Wars).

[31] Paes Dias M., Tripathi V., van der Heijden I. (2021). Loss of nuclear DNA ligase III reverts PARP inhibitor resistance in BRCA1/53BP1 double-deficient cells by exposing ssDNA gaps. Mol Cell.

[32] Hsiehchen D., Hsieh A., Samstein R.M. (2020). DNA repair gene mutations as predictors of immune checkpoint inhibitor response beyond tumor mutation burden. Cell Rep Med.

[33] Wang Y., Jiao X., Li S. (2021). Alterations in DNA damage response and repair genes as potential biomarkers for immune checkpoint blockade in gastrointestinal cancer. Cancer Biol Med.

[34] Guo J.A., Alshalalfa M., Kim D.Y. (2022). DNA repair and immune checkpoint blockade response. Cancer Genet.

[35] Zaenker P., Ziman M.R. (2013). Serologic autoantibodies as diagnostic cancer biomarkers--a review. Cancer Epidemiol Biomarkers Prev.

[36] Dong X., Lin D., Low C. (2013). Elevated expression of BIRC6 protein in non-small-cell lung cancers is associated with cancer recurrence and chemoresistance. J Thorac Oncol.

[37] Zhou J.H., Zhang B., Kernstine K.H., Zhong L. (2011). Autoantibodies against MMP-7 as a novel diagnostic biomarker in esophageal squamous cell carcinoma. World J Gastroenterol.

[38] Yang S.H., Liu C.T., Hong C.Q. (2021). Autoantibodies against p53, MMP-7, and Hsp70 as potential biomarkers for detection of nonmelanoma skin cancers. Dis Markers.

[39] Jiang T., Xie P., Liu H. (2016). Circulating anti-matrix metalloproteinase-7 antibodies may be a potential biomarker for oral squamous cell carcinoma. J Oral Maxillofac Surg.

[40] Li L.Y., Li E.M., Wu Z.Y. (2014). Overexpression of GRB2 is correlated with lymph node metastasis and poor prognosis in esophageal squamous cell carcinoma. Int J Clin Exp Pathol.

[41] Yu G.Z., Chen Y., Wang J.J. (2009). Overexpression of Grb2/HER2 signaling in Chinese gastric cancer: their relationship with clinicopathological parameters and prognostic significance. J Cancer Res Clin Oncol.

[42] Iwata T., Sedukhina A.S., Kubota M. (2021). A new bioinformatics approach identifies overexpression of GRB2 as a poor prognostic biomarker for prostate cancer. Sci Rep.

[43] Dou N., Yang D., Yu S., Wu B., Gao Y., Li Y. (2018). SNRPA enhances tumour cell growth in gastric cancer through modulating NGF expression. Cell Prolif.

[44] Zhang Y., Wang X., Wang H., Jiang Y., Xu Z., Luo L. (2022). Elevated small nuclear ribonucleoprotein polypeptide an expression correlated with poor prognosis and immune infiltrates in patients with hepatocellular carcinoma. Front Oncol.

[45] Roth E., Frohman M.A. (2018). Proliferative and metastatic roles for phospholipase D in mouse models of cancer. Adv Biol Regul.

[46] Liu B.W., Sun N., Lin H. (2023). The p53/ZEB1-PLD3 feedback loop regulates cell proliferation in breast cancer. Cell Death Dis.

[47] Lastwika K.J., Kunihiro A., Solan J.L. (2023). Posttranslational modifications induce autoantibodies with risk prediction capability in patients with small cell lung cancer. Sci Transl Med.

[48] Wang J., Cui B., Li X., Zhao X., Huang T., Ding X. (2023). The emerging roles of Hedgehog signaling in tumor immune microenvironment. Front Oncol.

[49] Rather T.B., Parveiz I., Bhat G.A. (2023). Colorectal cancer (CRC): investigating the expression of the suppressor of fused (SuFu) gene and its relationship with several inflammatory blood-based biomarkers. Biomedicines.

[50] Peris-Celda M., Carrion-Navarro J., Palacin-Aliana I. (2022). Suppressor of fused associates with dissemination patterns in patients with glioma. Front Oncol.

[51] Ullrich E., Bonmort M., Mignot G., Kroemer G., Zitvogel L. (2008). Tumor stress, cell death and the ensuing immune response. Cell Death Differ.

[52] Yi Y., Zhou Z., Shu S. (2012). Autophagy-assisted antigen cross-presentation: autophagosome as the argo of shared tumor-specific antigens and DAMPs. Oncoimmunology.

[53] Kroemer G., Galassi C., Zitvogel L., Galluzzi L. (2022). Immunogenic cell stress and death. Nat Immunol.

[54] Rafiq K., Bergtold A., Clynes R. (2002). Immune complex-mediated antigen presentation induces tumor immunity. J Clin Invest.

[55] Waight J.D., Chand D., Dietrich S. (2018). Selective FcγR co-engagement on APCs modulates the activity of therapeutic antibodies targeting T cell antigens. Cancer Cell.

[56] Chen X., Song X., Li K., Zhang T. (2019). FcγR-binding is an important functional attribute for immune checkpoint antibodies in cancer immunotherapy. Front Immunol.

[57] Teige I., Martensson L., Frendeus B.L. (2019). Targeting the antibody checkpoints to enhance cancer immunotherapy-focus on FcγRIIB. Front Immunol.

[58] Seidel U.J., Schlegel P., Lang P. (2013). Natural killer cell mediated antibody-dependent cellular cytotoxicity in tumor immunotherapy with therapeutic antibodies. Front Immunol.

[59] Wang W., Erbe A.K., Hank J.A., Morris Z.S., Sondel P.M. (2015). NK cell-mediated antibody-dependent cellular cytotoxicity in cancer immunotherapy. Front Immunol.

[60] Dixon K.J., Wu J., Walcheck B. (2021). Engineering anti-tumor monoclonal antibodies and Fc receptors to enhance ADCC by human NK cells. Cancers (Basel).

[61] Kanterman J., Sade-Feldman M., Baniyash M. (2012). New insights into chronic inflammation-induced immunosuppression. Semin Cancer Biol.

[62] Soussan S., Pupier G., Cremer I. (2024). Unraveling the complex interplay between anti-tumor immune response and autoimmunity mediated by B cells and autoantibodies in the era of anti-checkpoint monoclonal antibody therapies. Front Immunol.

[63] Wang T., Qin Y., Lai H. (2019). The prognostic value of ADRA1 subfamily genes in gastric carcinoma. Oncol Lett.

[64] Wallukat G., Jandrig B., Becker N.P. (2020). Autoantibodies directed against alpha1-adrenergic receptor and endothelin receptor A in patients with prostate cancer. Auto Immun Highlights.

[65] Stalin J., Nollet M., Dignat-George F., Bardin N., Blot-Chabaud M. (2017). Therapeutic and diagnostic antibodies to CD146: thirty years of research on its potential for detection and treatment of tumors. Antibodies (Basel).

[66] Mills L., Tellez C., Huang S. (2002). Fully human antibodies to MCAM/MUC18 inhibit tumor growth and metastasis of human melanoma. Cancer Res.

