# Supplementary material for: Autoantibody profiling to predict response to the anti-PD-1 therapy, pembrolizumab, in rare tumors
Source: ESMO Open. 2025 Aug 5;10(8):105518. doi: 10.1016/j.esmoop.2025.105518 (PMC12345252; doi:10.1016/j.esmoop.2025.105518)
Supplement: Supplementary Material [file mmc1.docx]

**Supplementary Materials**


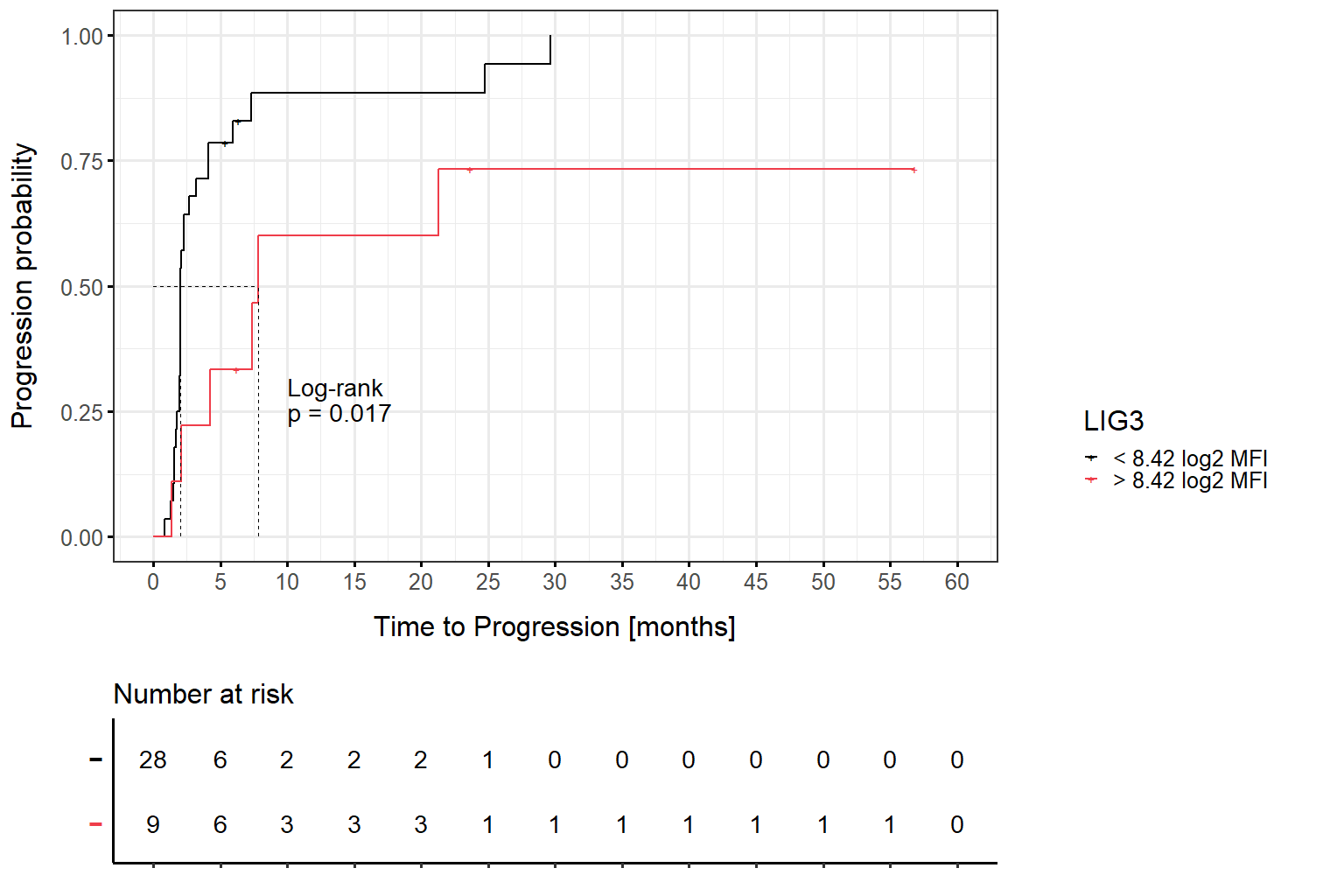

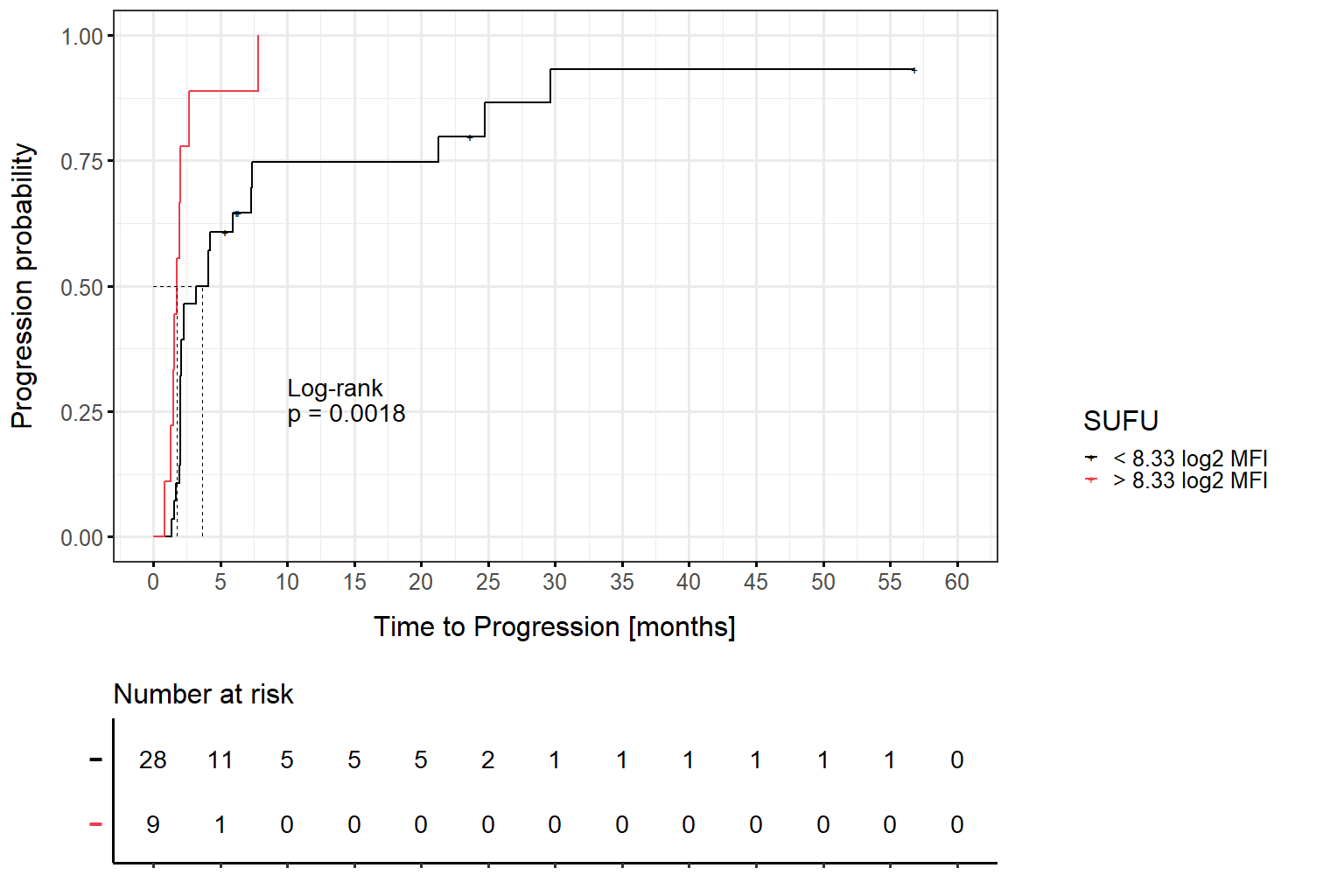

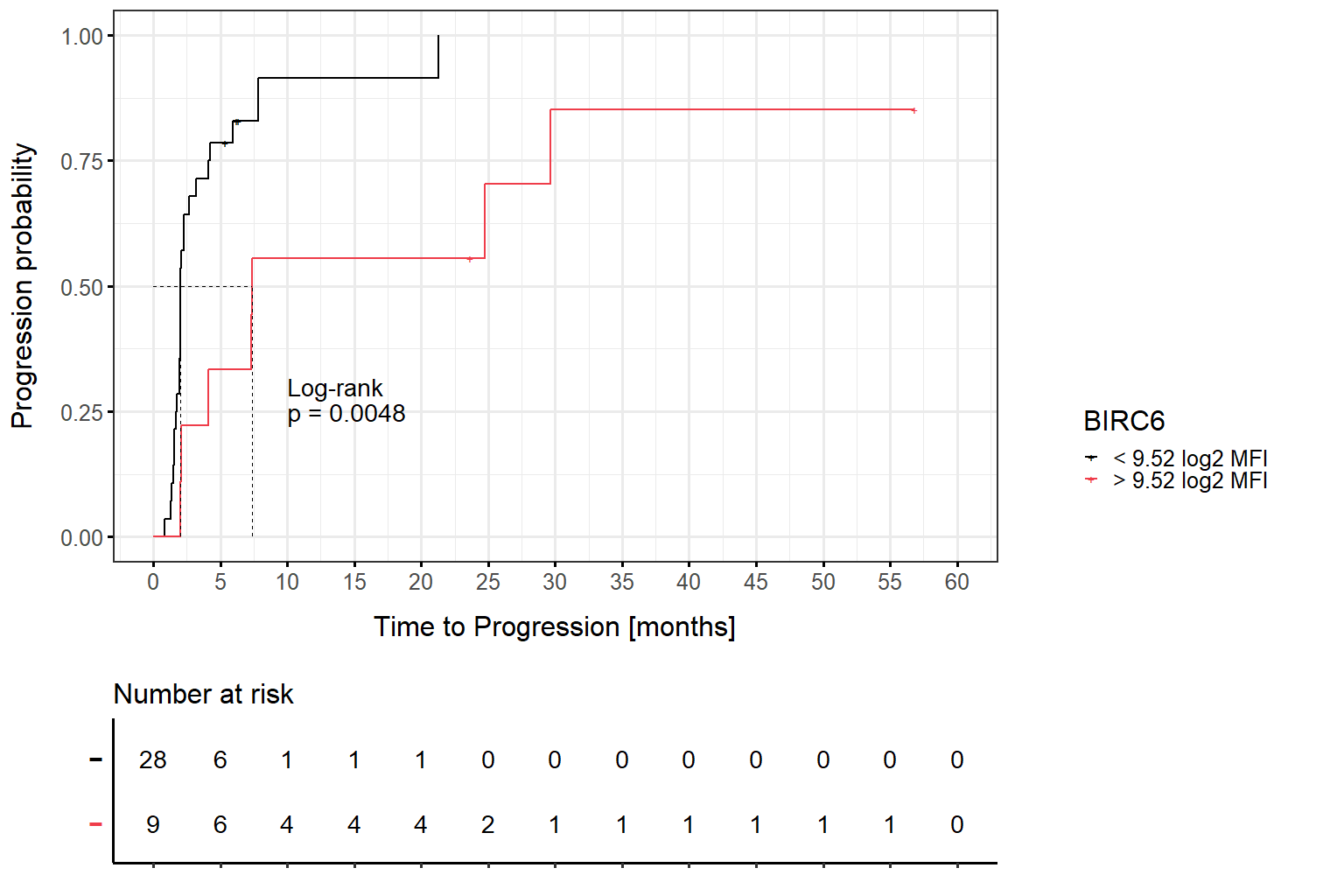

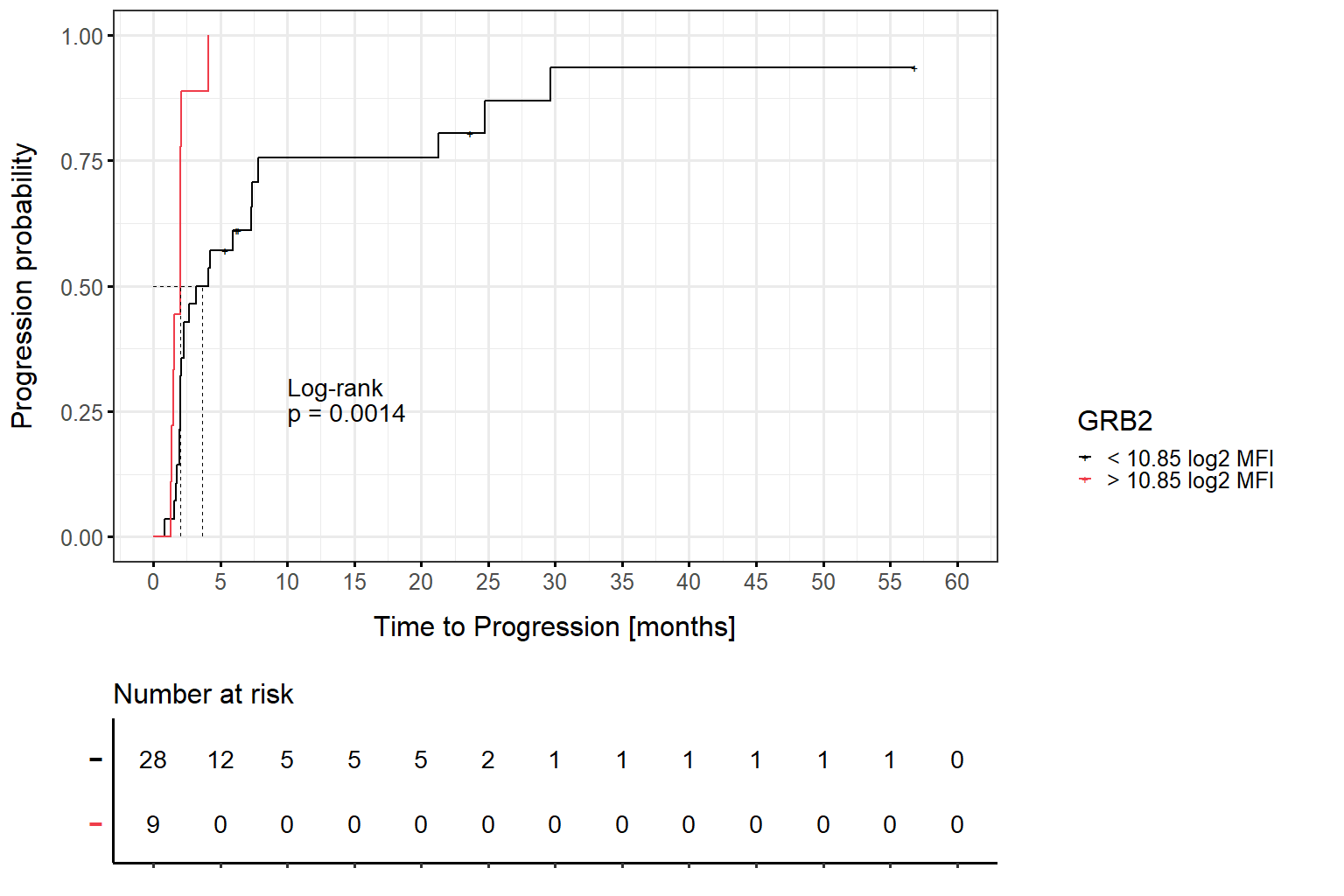

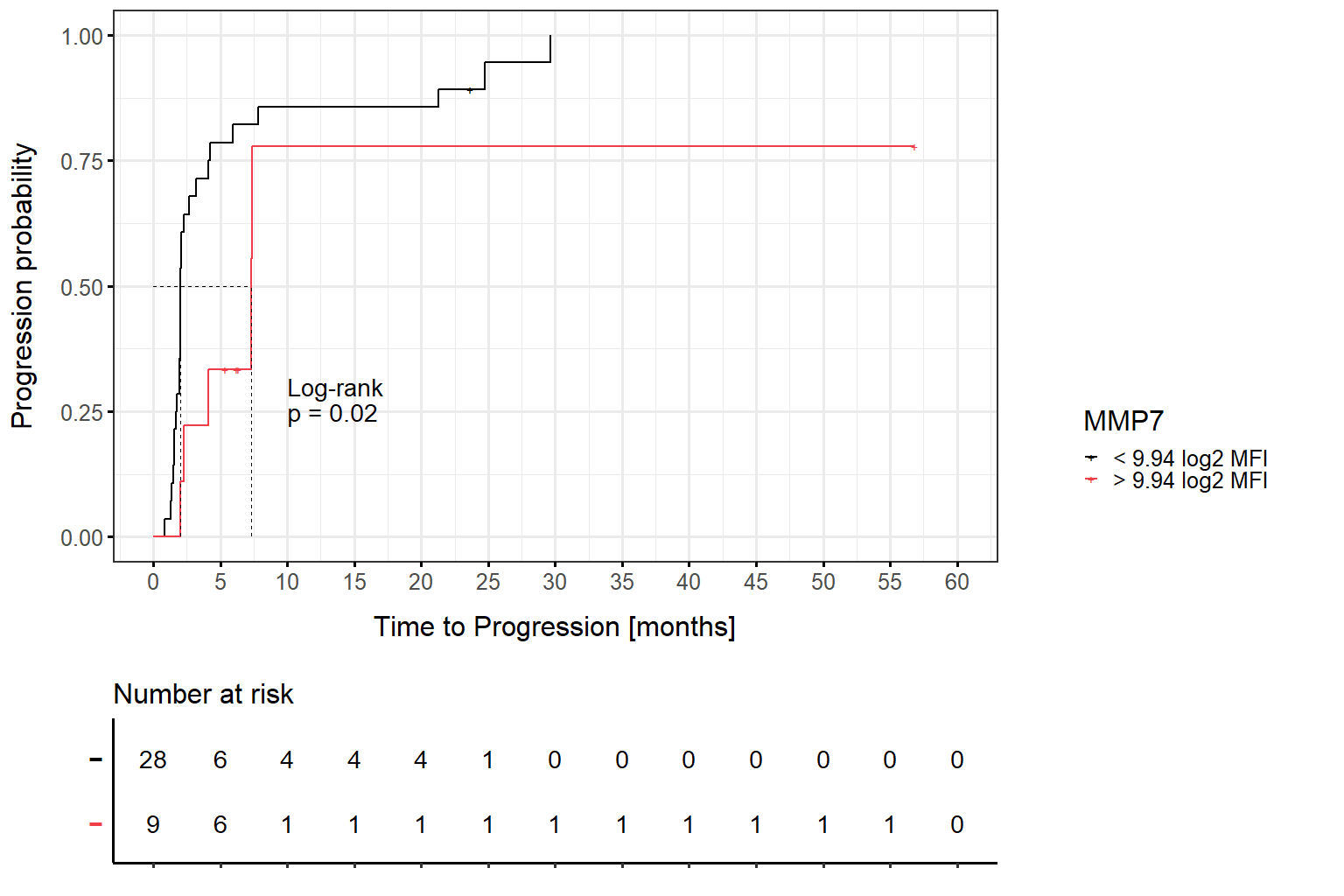


**Fig. S1. Autoantibodies associated with time to progression.**

Kaplan-Meier estimates of time to progression for patients with rare tumors stratified by autoantibody level. P-values were calculated using the log-rank test.


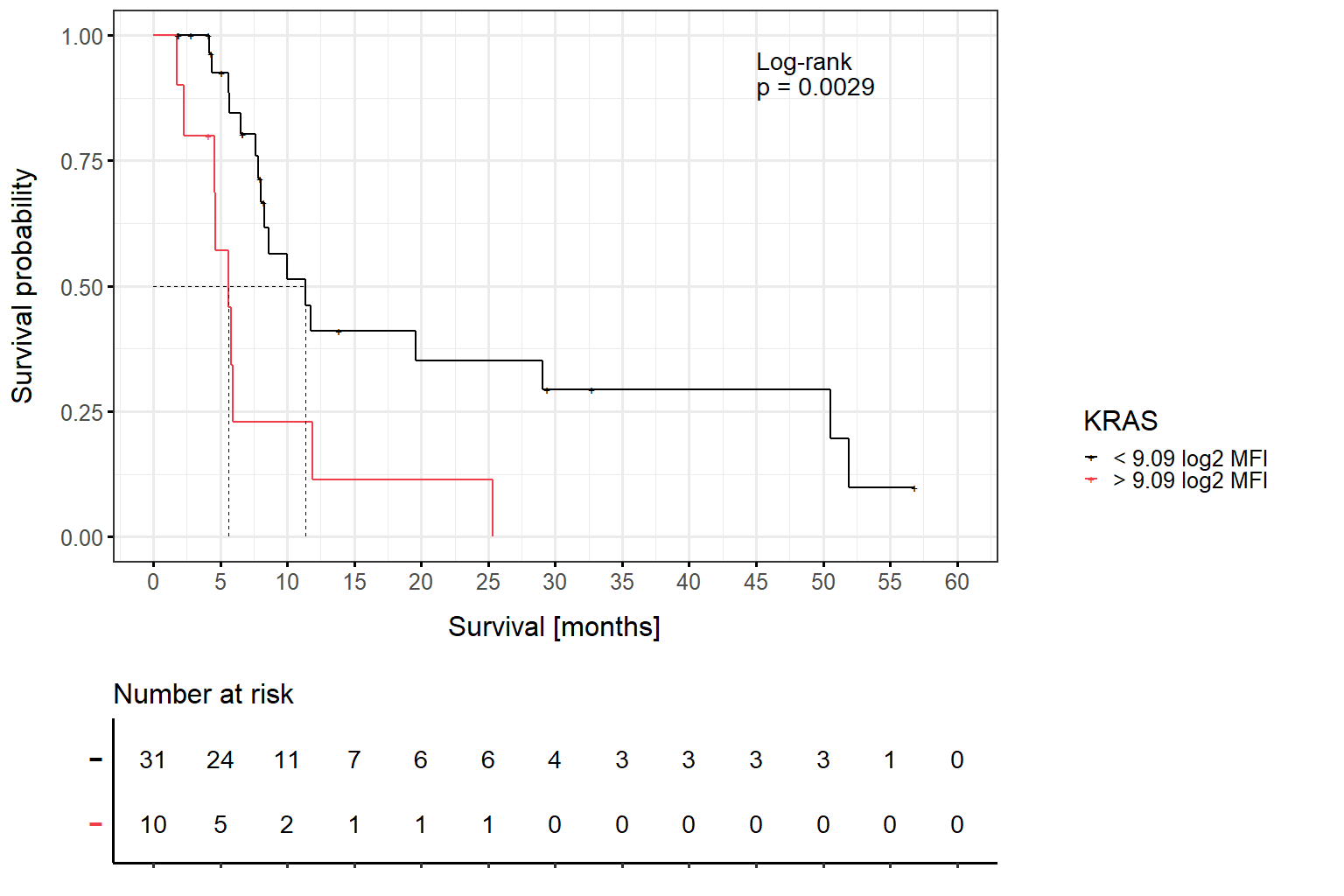

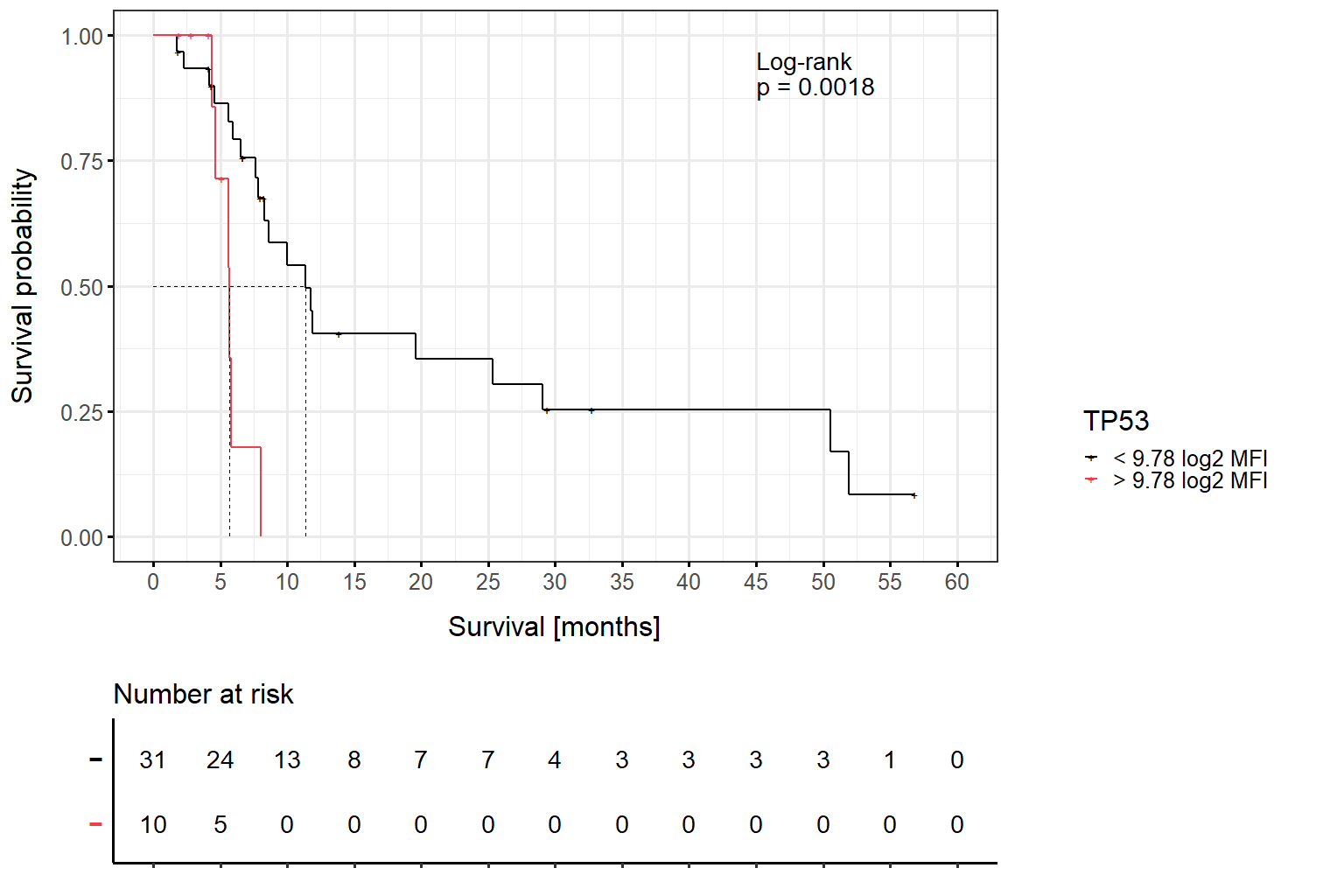

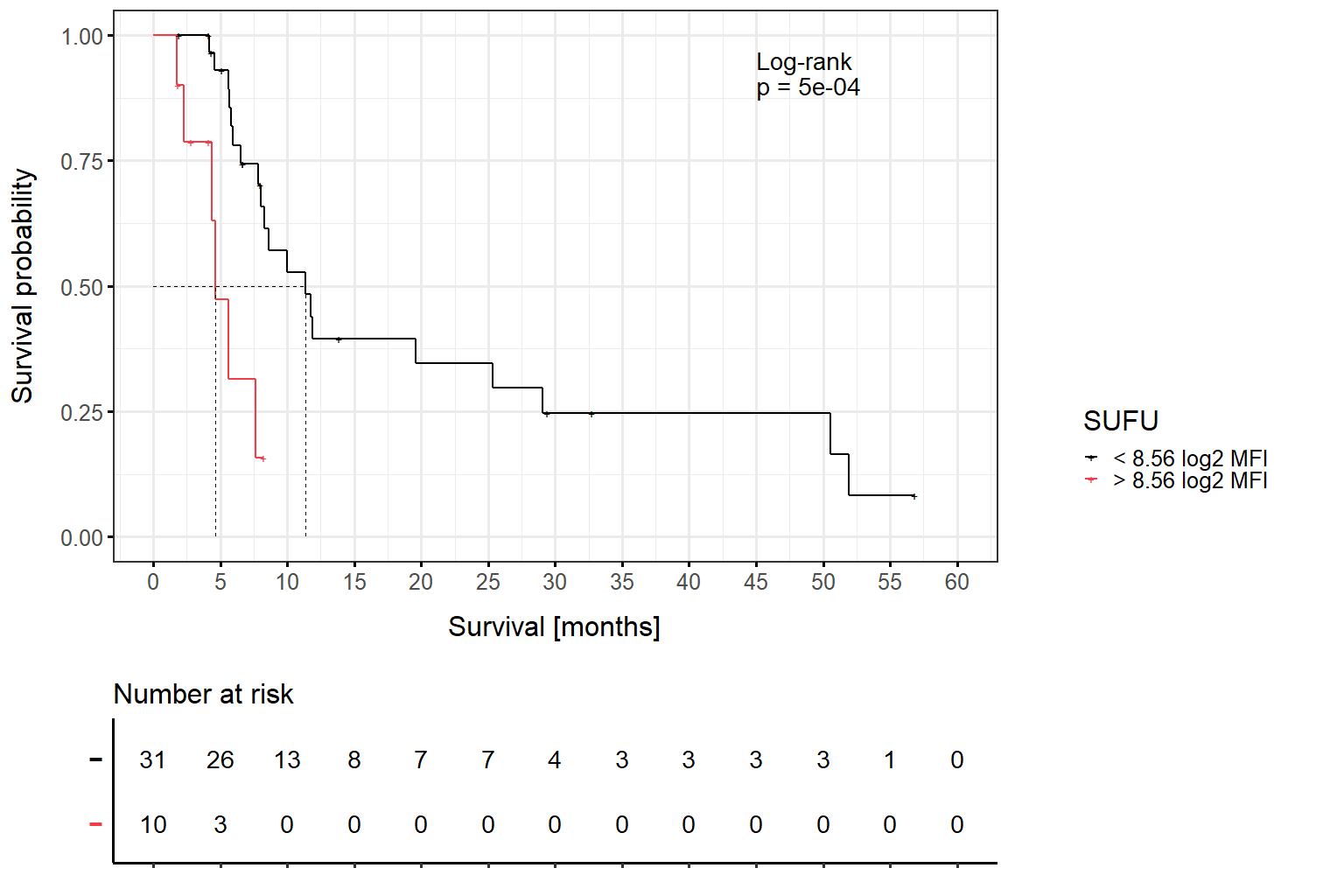

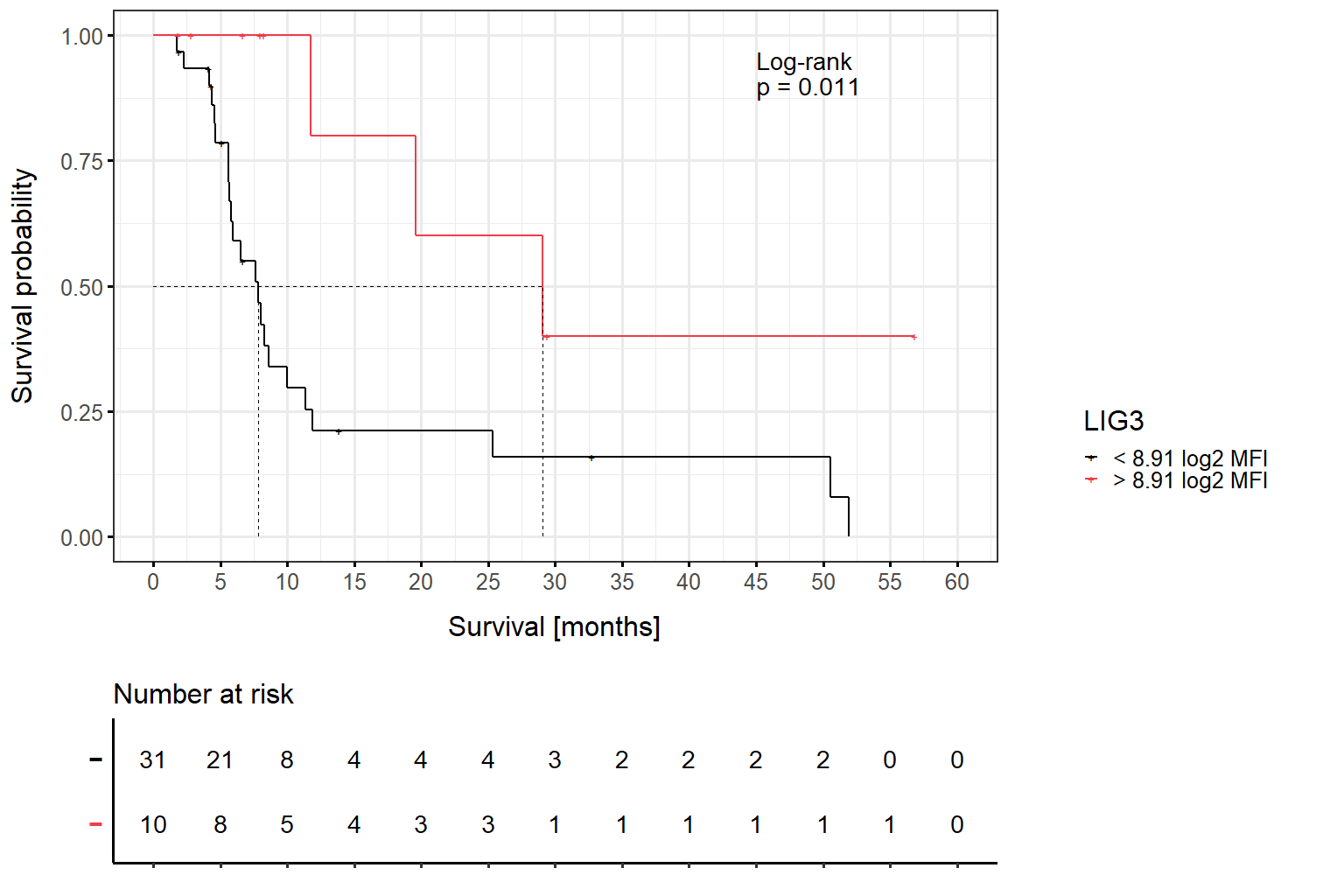

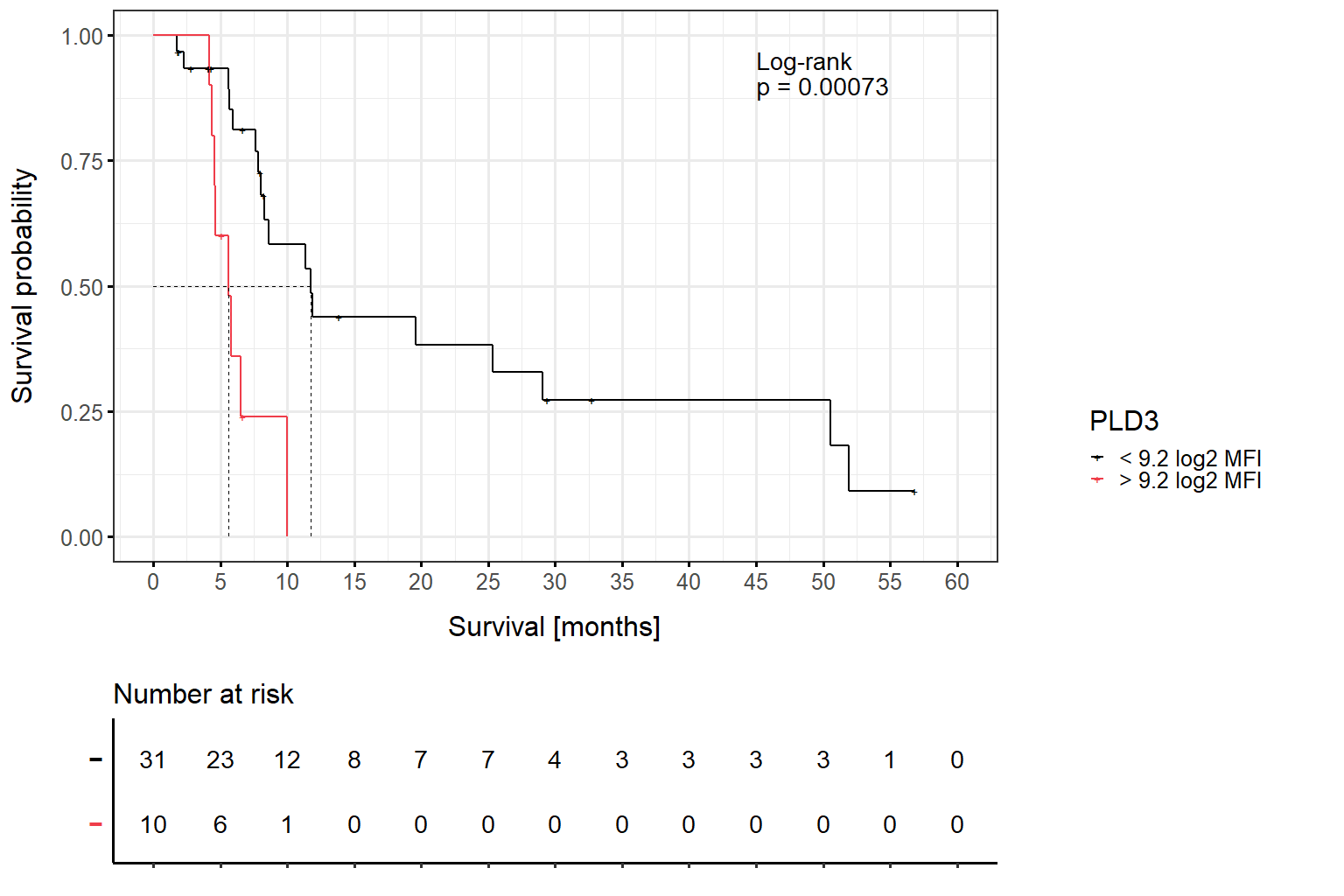


**Fig. S2. Autoantibodies associated with overall survival.**

Kaplan-Meier estimates of overall survival for patients with rare tumors stratified by autoantibody level. P-values were calculated using the log-rank test.

**Table S1. Positive autoantibodies in patients with rare tumors vs. healthy controls.**

| **Antigen** | **D-score** | **Fold-change** | **P-value** |
| --- | --- | --- | --- |
| **NRG1** | 2.837 | 1.480 | 0.001 |
| **RGS5** | 2.755 | 1.564 | 0.001 |
| **TRIM21** | 2.754 | 1.789 | 0.001 |
| **APC** | 2.524 | 1.908 | 0.002 |
| **IFNA1** | 2.488 | 1.765 | 0.002 |
| **SOST** | 2.326 | 1.925 | 0.004 |
| **POLE4** | 2.304 | 1.678 | 0.004 |
| **MYO1D** | 2.298 | 1.743 | 0.004 |
| **IFNA5** | 2.288 | 1.629 | 0.004 |
| **VEGFA** | 2.282 | 1.466 | 0.004 |
| **RO60** | 2.125 | 1.425 | 0.007 |
| **IFNA16** | 2.064 | 1.395 | 0.009 |
| **IL12AB** | 2.028 | 1.650 | 0.010 |
| **APOBEC3G** | 2.024 | 1.293 | 0.010 |

Note: D-scores, fold-changes, and p-values were calculated from a significance analysis of microarrays

**Table S2. Autoantibodies associated with clinical benefit.**

| **Antigen** | **D-score** | **Fold-change** | **P-value** |
| --- | --- | --- | --- |
| **LIG3** | 4.065 | 2.473 | 0.001 |
| **BIRC6** | 2.968 | 3.528 | 0.002 |
| **SYCE1** | 2.961 | 3.905 | 0.002 |
| **RBBP5** | 2.785 | 2.934 | 0.003 |
| **DNAJC2** | 2.458 | 2.1 | 0.006 |
| **THOP1** | 2.334 | 1.859 | 0.008 |
| **MYLK** | 2.149 | 2.819 | 0.013 |
| **PNMA6A** | 2.01 | 2.549 | 0.019 |

Note: D-scores, fold-changes, and p-values were calculated from a significance analysis of microarrays.

**Table S3. Autoantibodies associated with time to progression (TTP).**

| **Antigen** | **HR (95% CI)*** | **Log2 HR (95% CI)*** | **P-value^†^** | **P-value^‡^** |
| --- | --- | --- | --- | --- |
| **Associated with longer TTP** |  |  |  |  |
| **MMP7** | 0.24 (0.07, 0.78) | -2.05 (-3.75, -0.36) | 0.018 | 0.020 |
| **PRKCA** | 0.46 (0.27, 0.8) | -1.11 (-1.9, -0.33) | 0.006 | 0.008 |
| **LIG3** | 0.46 (0.27, 0.79) | -1.11 (-1.89, -0.33) | 0.005 | 0.017 |
| **RPS6KA1** | 0.65 (0.43, 0.98) | -0.62 (-1.21, -0.03) | 0.038 | 0.035 |
| **BIRC6** | 0.67 (0.5, 0.9) | -0.58 (-1, -0.16) | 0.007 | 0.005 |
| **Associated with shorter TTP** |  |  |  |  |
| **GRB2** | 4.2 (1.17, 15.16) | 2.07 (0.22, 3.92) | 0.028 | 0.001 |
| **SUFU** | 2.36 (1.48, 3.79) | 1.24 (0.56, 1.92) | 0.0005 | 0.002 |
| **ANGPT2** | 2.21 (1.31, 3.73) | 1.14 (0.38, 1.9) | 0.003 | 0.000 |
| **MSH2** | 2.19 (1.14, 4.24) | 1.13 (0.18, 2.08) | 0.019 | 0.005 |
| **ADRA1A** | 2.04 (1.17, 3.57) | 1.03 (0.23, 1.84) | 0.012 | 0.016 |
| **NRG1** | 1.99 (1.16, 3.41) | 0.99 (0.21, 1.77) | 0.013 | 0.001 |
| **RGL2** | 1.92 (1.23, 2.99) | 0.94 (0.29, 1.58) | 0.004 | 0.014 |
| **DFFA** | 1.87 (1.27, 2.74) | 0.9 (0.35, 1.46) | 0.001 | 0.007 |
| **GNAI2** | 1.84 (1.3, 2.62) | 0.88 (0.38, 1.39) | 0.001 | 0.002 |
| **BCL2L1** | 1.79 (1.17, 2.73) | 0.84 (0.23, 1.45) | 0.007 | 0.041 |
| **TGFBR2** | 1.79 (1.14, 2.8) | 0.84 (0.19, 1.49) | 0.012 | 0.021 |
| **TOLLIP** | 1.78 (1.19, 2.68) | 0.83 (0.25, 1.42) | 0.005 | 0.021 |
| **PTPRR** | 1.75 (1.14, 2.68) | 0.81 (0.2, 1.42) | 0.010 | 0.005 |
| **IGF2BP3** | 1.63 (1.06, 2.51) | 0.71 (0.09, 1.33) | 0.025 | 0.028 |
| **CDH1** | 1.58 (1.17, 2.15) | 0.66 (0.22, 1.1) | 0.003 | 0.045 |
| **TNFAIP3** | 1.55 (1.04, 2.31) | 0.63 (0.05, 1.2) | 0.033 | 0.033 |
| **HMMR** | 1.51 (1.11, 2.05) | 0.6 (0.15, 1.04) | 0.008 | 0.001 |

Abbreviations: HR, hazard ratio; CI, confidence interval.

*HRs and 95% CIs were from a Cox proportional hazards model.

**^†^**P-values were from Cox regression tests.

**^‡^**P-values were from log-rank tests.

**Table S4. Autoantibodies associated with overall survival (OS).**

| **Antigen** | **HR (95% CI)*** | **Log2 HR (95% CI)*** | **P-value^†^** | **P-value^‡^** |
| --- | --- | --- | --- | --- |
| **Associated with longer OS** |  |  |  |  |
| **SNRPA** | 0.19 (0.07, 0.57) | -2.37 (-3.92, -0.82) | 0.003 | 0.009 |
| **LIG3** | 0.48 (0.27, 0.83) | -1.07 (-1.88, -0.26) | 0.010 | 0.011 |
| **MCAM** | 0.52 (0.3, 0.88) | -0.96 (-1.73, -0.18) | 0.016 | 0.012 |
| **RGS5** | 0.54 (0.31, 0.95) | -0.88 (-1.67, -0.08) | 0.031 | 0.029 |
| **STMN1** | 0.67 (0.49, 0.92) | -0.58 (-1.03, -0.13) | 0.012 | 0.003 |
| **Associated with shorter OS** |  |  |  |  |
| **PLD3** | 4.29 (1.66, 11.07) | 2.1 (0.74, 3.47) | 0.003 | 0.001 |
| **PTPRA** | 3.53 (1.41, 8.81) | 1.82 (0.5, 3.14) | 0.007 | 0.000 |
| **KRAS** | 2.75 (1.25, 6.05) | 1.46 (0.33, 2.6) | 0.012 | 0.003 |
| **PTPRR** | 2.61 (1.42, 4.79) | 1.38 (0.51, 2.26) | 0.002 | 0.002 |
| **SUFU** | 2.56 (1.43, 4.59) | 1.36 (0.52, 2.2) | 0.002 | 0.000 |
| **ST3GAL4** | 2.12 (1.08, 4.17) | 1.08 (0.11, 2.06) | 0.030 | 0.022 |
| **ATP1B3** | 1.98 (1.12, 3.51) | 0.99 (0.17, 1.81) | 0.019 | 0.010 |
| **TP53** | 1.79 (1.01, 3.17) | 0.84 (0.02, 1.67) | 0.045 | 0.002 |
| **ADRA1A** | 1.76 (1.07, 2.9) | 0.82 (0.09, 1.54) | 0.027 | 0.033 |
| **NRG1** | 1.74 (1.12, 2.72) | 0.8 (0.16, 1.44) | 0.014 | 0.000 |
| **TOLLIP** | 1.68 (1.06, 2.67) | 0.75 (0.08, 1.42) | 0.028 | 0.012 |
| **RPLP2** | 1.59 (1.18, 2.13) | 0.67 (0.24, 1.09) | 0.002 | 0.0029 |

Abbreviations: HR, hazard ratio; CI, confidence interval.

*HRs and 95% CIs were from a Cox proportional hazards model.

**^†^**P-values were from Cox regression tests.

**^‡^**P-values were from log-rank tests.
